# Clinical Characteristics and Thyroid Findings in Children with Isolated Premature Adrenarche

**DOI:** 10.3390/children13091221

**Published:** 2026-09-09

**Authors:** Sibel Aka, Seymanur Kocyigit, Saygin Abali, Serap Semiz

**Affiliations:** 1 Department of Pediatrics, Acibadem Dr. Sinasi Can Hospital, Istanbul 34718, Turkey; sibel.aka@acibadem.com; 2 Department of Pediatrics, School of Medicine, Acibadem Mehmet Ali Aydinlar University, Istanbul 34638, Turkey; 3 Division of Pediatric Endocrinology, Department of Pediatrics, School of Medicine, Acibadem Mehmet Ali Aydinlar University, Istanbul 34638, Turkey

**Keywords:** premature adrenarche, adrenal, nonclassical congenital adrenal hyperplasia, thyroid, autoimmunity

## Abstract

**Highlights:**

**What are the main findings?**

• Children with idiopathic premature adrenarche frequently showed accelerated linear growth and excess weight, while lower birth weight was associated with higher adrenal androgen secretion.
• Thyroid function parameters were not associated with adrenal androgen secretion; thyroid autoantibody findings were limited to a selectively tested subgroup.

**What are the implications of the main findings?**

• The association between lower birth weight and higher DHEAS levels supports a potential role of prenatal growth in subsequent adrenal maturation.
• The thyroid findings should be interpreted cautiously and warrant confirmation in prospective controlled studies with systematic thyroid autoantibody assessment.

**Abstract:**

**Objectives**: Premature adrenarche (PA) is associated with accelerated adrenal maturation, but data regarding thyroid characteristics in children with isolated PA are limited. We aimed to describe the clinical features of children with idiopathic PA and investigate the relationships between thyroid function, thyroid autoimmunity, and adrenal androgen secretion. **Methods**: This retrospective single-center study included 435 children (378 girls, 57 boys) with idiopathic PA evaluated between 2014 and 2024. Clinical, anthropometric, biochemical, and thyroid data were analyzed. Associations between adrenal and thyroid parameters were examined using correlation and group comparison analyses. **Results**: Girls accounted for 86.9% of the cohort. Children were generally taller than their genetic target height and frequently had excess weight. Boys exhibited higher serum dehydroepiandrosterone sulfate (DHEAS) concentrations than girls and more often met criteria for exaggerated adrenarche despite less advanced clinical androgenization. Lower birth weight (BW) was associated with higher DHEAS concentrations, and children born with low BW more frequently demonstrated exaggerated adrenarche. BW SDS showed a significant inverse correlation with DHEAS concentrations. In contrast, DHEAS concentrations were unrelated to body mass index (BMI) SDS or bone age advancement. Height SDS increased progressively with increasing BMI category in both sexes, whereas DHEAS concentrations remained comparable across weight groups. Thyroid abnormalities were identified in 8.0% of the cohort. Free thyroxine and thyroid-stimulating hormone concentrations were within the normal range in most children and showed no association with DHEAS concentrations or exaggerated adrenarche status. Although thyroid autoantibody positivity was frequent among the tested subgroup, it was not associated with adrenal androgen concentrations. **Conclusions**: Idiopathic PA is characterized by accelerated linear growth, frequent excess weight, and an inverse relationship between birth weight and adrenal androgen secretion. Thyroid function was not associated with adrenal androgen production within this PA cohort. The frequent thyroid autoantibody positivity observed in the selected tested subgroup should be interpreted cautiously and cannot be considered PA-specific in the absence of healthy control groups; prospective controlled studies are needed.

## 1. Introduction

Adrenarche is a unique maturational process marked by a progressive increase in adrenal androgen production, driven by the development and functional maturation of the adrenal zona reticularis [1,2]. It is biochemically characterized by rising concentrations of dehydroepiandrosterone (DHEA), dehydroepiandrosterone sulfate (DHEAS), and other adrenal androgen precursors, independent of activation of the hypothalamic–pituitary–gonadal axis [3,4]. Clinically, adrenarche manifests as the appearance of pubic and axillary hair, acne, oily skin and hair, and adult-type body odor [1,2].

Premature adrenarche (PA) is defined as the onset of these manifestations before age 8 in girls and before age 9 in boys [1,5]. Although PA was traditionally considered part of normal developmental variation, accumulating evidence suggests that it may be linked to unfavorable metabolic and endocrine outcomes. Previous studies have demonstrated associations between PA and low birth weight, small-for-gestational-age (SGA) birth, accelerated childhood growth, obesity, insulin resistance, and components of the metabolic syndrome [6]. Furthermore, longitudinal data suggest that girls with PA may exhibit an increased risk of functional ovarian hyperandrogenism and polyendocrine metabolic ovarian syndrome (PMOS) later in adolescence and adulthood [6,7,8]. These observations support the concept that PA may represent an early marker of broader alterations in endocrine and metabolic regulation rather than an isolated developmental variant.

Thyroid hormones are fundamental regulators of growth, development, and energy homeostasis. Beyond their established effects, emerging evidence indicates a close physiological interaction between the thyroid and adrenal axes. Experimental studies have demonstrated expression of thyroid hormone receptor-β within the developing adrenal cortex [9]. Congenital hypo- and hyperthyroidism have been shown to alter adrenal morphology, gene expression, and steroid hormone production, supporting a mechanistic link between thyroid status and adrenal function. Thyroid-stimulating hormone (TSH) concentrations have been reported to correlate positively with adrenal steroid secretion in pediatric populations [10]. Evidence from longitudinal population-based studies indicates that children with higher thyroid hormone levels tend to enter puberty earlier, raising the possibility that thyroid hormones may contribute to the regulation of developmental timing [11,12]. Despite emerging data, thyroid status has not been systematically evaluated in large cohorts of children with isolated PA, and the relationships among adrenal androgen excess, thyroid function, thyroid autoimmunity, and clinical characteristics remain poorly understood.

In this study, we aimed to comprehensively characterize the clinical features of a large cohort of children with isolated PA and to investigate thyroid function and thyroid autoimmunity and their associations with anthropometric, hormonal, and biochemical markers of adrenarche.

## 2. Materials and Methods

### 2.1. Study Design and Participants

**Figure 1:**
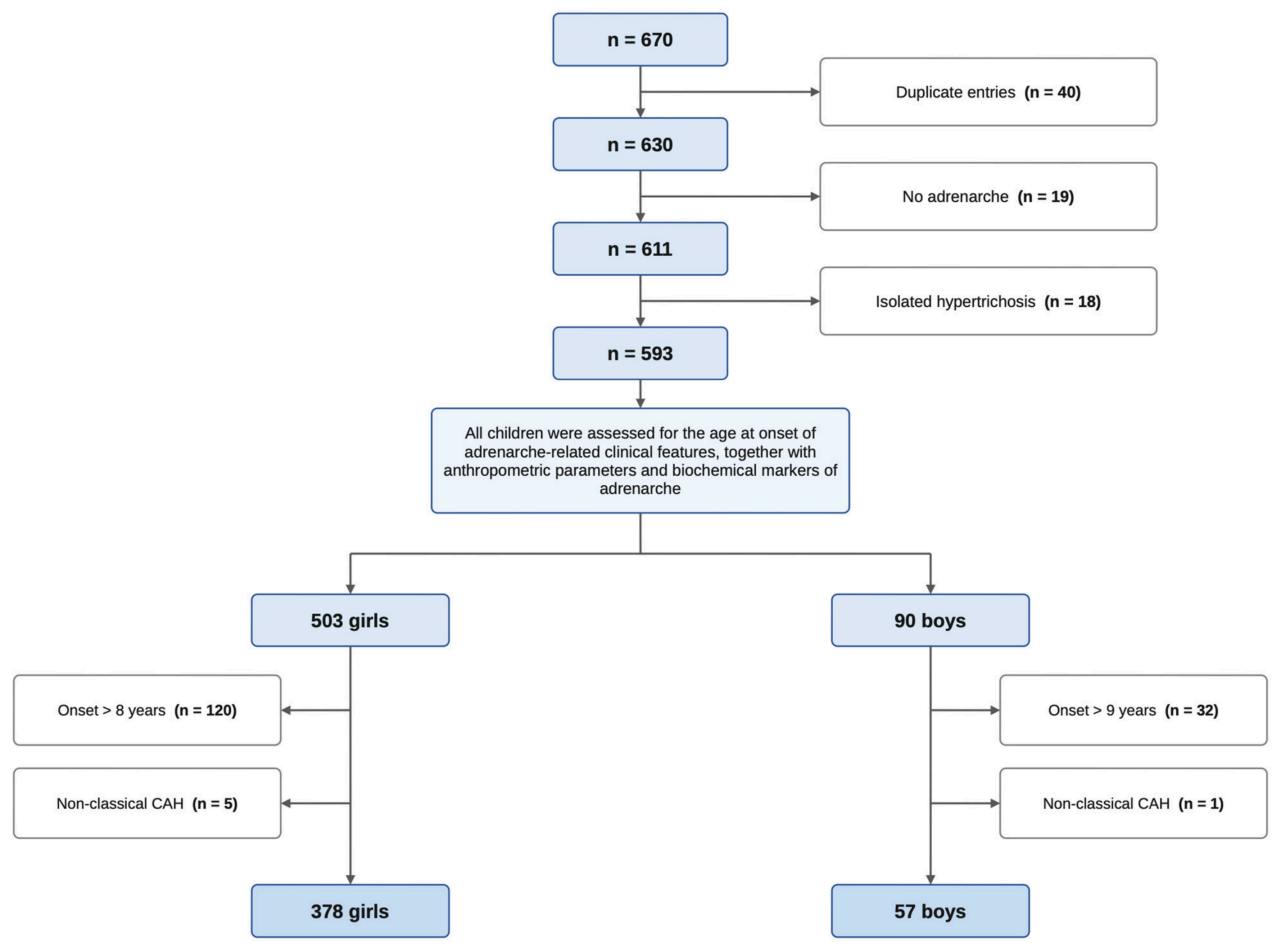
Flowchart of participant selection and study cohort derivation.

This single-center retrospective descriptive study initially evaluated 670 records of children who presented to the Pediatric Endocrinology Unit between 2014 and 2024 with suspected PA. The patient selection process is summarized in Figure 1.

Among the 670 screened records, duplicate entries, children without evidence of adrenarche, those with isolated hypertrichosis, and those whose adrenarche-related signs developed beyond the established age thresholds (>8 years in girls and >9 years in boys) were excluded. The remaining 441 children (383 girls and 58 boys) underwent comprehensive clinical and biochemical evaluation.

Following this assessment, five girls (5/383, 1.3%) and one boy (1/58, 1.7%) were diagnosed with non-classical congenital adrenal hyperplasia (NC-CAH) and were subsequently excluded from the analysis. Consequently, a total of 435 patients (378 girls and 57 boys) with isolated idiopathic PA were included in the final study cohort.

### 2.2. Data Collection

Demographic and clinical data were retrieved from electronic medical records, including date of birth, date of presentation, sex, duration of adrenarche-related signs, gestational age, birth weight (BW), height, weight, body mass index (BMI), parental heights, presence of goitre on physical examination, presence of axillary hair, Tanner stage of pubic hair, Tanner stage of breast development in girls, and genital stage in boys. Bone age (BA) was assessed using the Greulich and Pyle method by the same experienced pediatric endocrinologist (SS) and recorded for all patients. BW, height, weight, and BMI standard deviation scores (SDS) were calculated using Turkish reference data [13,14]. Low BW (LBW) was defined as a BW below 2500 g, irrespective of gestational age, according to World Health Organization criteria. SGA was defined as a BW SDS below −2.0 for gestational age and sex [15].

### 2.3. Hormonal Evaluation

Twelve-hour fasting venous blood samples were obtained for measurement of DHEAS, 17-hydroxyprogesterone (17-OHP), androstenedione, and total testosterone. Serum DHEAS and total testosterone concentrations were measured using chemiluminescence immunoassay (CLIA) on the Siemens Atellica Solution platform (Siemens Healthcare Diagnostics, Erlangen, Germany). Serum 17-OHP and androstenedione concentrations were determined by liquid chromatography–tandem mass spectrometry (LC–MS/MS) using the Agilent analytical platform (Agilent Technologies, Santa Clara, CA, USA).

To evaluate the potential impact of the severity of adrenal androgen excess, children with serum DHEAS concentrations > 130 µg/dL were classified as having exaggerated adrenarche, whereas those with DHEAS concentrations ≤ 130 µg/dL were classified as having non-exaggerated adrenarche [1]. Children with baseline 17-OHP concentrations > 2 ng/mL underwent a standard 250-μg adrenocorticotropic hormone (ACTH) stimulation test to exclude NC-CAH.

Serum free thyroxine (fT4) and thyroid-stimulating hormone (TSH) concentrations were measured in all participants. In children with goitre on physical examination and/or elevated TSH concentrations, thyroid autoantibodies, including anti-thyroid peroxidase (anti-TPO) and anti-thyroglobulin (anti-Tg), were assessed (*n* = 14). Additionally, retrospective review of the medical records identified 23 children without evidence of thyroid abnormalities in whom thyroid autoantibodies had also been assessed. Serum fT4, TSH, anti-TPO, and anti-Tg concentrations were measured using CLIA on the Siemens Atellica Solution platform (Siemens Healthcare Diagnostics, Erlangen, Germany).

Ethical approval for this retrospective study was granted by the Ethics Committee of Acibadem Mehmet Ali Aydinlar University (2025-01/11), and the study was conducted in accordance with the principles of the Declaration of Helsinki. As no interventions were performed, individual informed consent from patients or their parents was not required, and this requirement was formally waived by the ethics committee.

### 2.4. Statistical Analysis

Statistical analyses were performed using NCSS Statistical Software (version 2020; NCSS LLC, Kaysville, UT, USA). Generative artificial intelligence tools, including ChatGPT (GPT-4o; OpenAI, San Francisco, CA, USA) and Claude (Claude 3.5 Sonnet; Anthropic, San Francisco, CA, USA), were used to assist with statistical workflow development, data exploration, and interpretation of statistical outputs. All analyses, verification of results, and interpretation of findings were independently performed and validated by the authors, who take full responsibility for the accuracy and integrity of the reported results.

Continuous variables were assessed for normality using the Shapiro–Wilk test and visual inspection of box plots. Data are presented as mean ± standard deviation (SD) for normally distributed variables and median (interquartile range [IQR]) for non-normally distributed variables. Categorical variables are presented as frequencies and percentages.

Comparisons between two independent groups were performed using the Student’s *t*-test for normally distributed variables and the Mann–Whitney U test for non-normally distributed variables. Comparisons among more than two groups were conducted using the Kruskal–Wallis test, followed by Dunn–Bonferroni post hoc analyses when appropriate. Categorical data were analyzed using either the chi-square test or Fisher’s exact test, as appropriate.

Relationships between continuous variables were assessed with Pearson’s correlation for normally distributed data and Spearman’s rank correlation for data that did not meet normality assumptions. When appropriate, DHEAS concentrations were log-transformed to evaluate whether the observed associations were robust to transformation. All statistical tests were two-tailed, with statistical significance defined as *p* < 0.05.

## 3. Results

This single-center retrospective descriptive study evaluated children referred for suspected PA. During the 10-year study period, 630 unique children were referred. Following diagnostic assessment, 30.0% (189/630) were excluded because they did not fulfill the diagnostic criteria for PA. Among children presenting with PA, the prevalence of NC-CAH was 1.3% of girls and 1.7% of boys.

### 3.1. Clinical and Biochemical Characteristics

A total of 435 children with idiopathic PA were included in the study, comprising 378 girls (86.9%) and 57 boys (13.1%) (female/male ratio was 6.6:1). The median chronological age (CA) at presentation was 7.8 (7.0–8.4) years in girls and 8.3 (7.7–9.1) years in boys. Gestational age and BW SDS were generally unremarkable in both sexes. Prematurity was observed in 9.8% of girls and 10.5% of boys, whereas 4.8% of girls were born SGA, with no SGA cases among boys (χ^2^ = 0.65, *p* = 0.420). The LBW ratio in our cohort was 17.2%.

The clinical and biochemical features of girls and boys are presented in Table 1.

Age at presentation was comparable between children with LBW and those with birth weights ≥ 2500 g, and BW SDS was not associated with age at presentation (Table 2).

At presentation, children demonstrated higher height SDS for their parental target, with median height SDS values of 0.9 (0.2–1.7) in girls and 1.0 (0.1–2.1) in boys, corresponding to median height SDS–midparental height (MPH) SDS differences of 0.8 (0.0–1.5) and 1.0 (0.5–1.8), respectively.

Median BMI SDS was 0.8 (0.1–1.5) in girls and 0.9 (0.1–1.5) in boys. Obesity was present in 20.9% of girls and 19.3% of boys, while overweight status was observed in 20.6% and 22.8%, respectively.

Most children presented with Tanner stage 2 pubic hair development, accounting for 62.7% of girls and 77.2% of boys. Pubic hair stage distribution also differed significantly between sexes (χ^2^ = 6.51, *p* = 0.039), with a greater proportion of boys in Tanner stage 2 and a lower proportion in stage 3. Axillary hair was present in approximately half of the girls (51.8%) and in 38.6% of boys (χ^2^ = 4.42, *p* = 0.110).

Bone age (BA) was mildly advanced in girls, with a median BA–CA difference of 0.5 (−0.1 to 1.4) years, whereas boys showed no meaningful advancement [−0.2 (−0.3 to 0.3) years].

Biochemically, serum DHEAS concentrations were elevated in both sexes and were higher in boys than girls [113.5 (69.7–142.8) vs. 85.0 (55.5–115.0) µg/dL] (*p* = 0.003). Overall, 21.4% of children met the criteria for exaggerated adrenarche. Exaggerated adrenarche was more common in boys than in girls, occurring in 37.5% and 18.8% of cases, respectively (χ^2^ = 7.53, *p* = 0.006). Median androstenedione concentrations were comparable between sexes, measuring 0.28 (0.18–0.47) ng/mL in boys and 0.26 (0.15–0.38) ng/mL in girls (*p* = 0.192). Serum DHEAS concentrations demonstrated a moderate positive correlation with androstenedione levels (Spearman ρ = 0.386, *p* < 0.001). Sex-stratified analyses yielded similar findings, with significant positive correlations observed in both girls (ρ = 0.372, *p* < 0.001) and boys (ρ = 0.487, *p* < 0.001), indicating concordant increases in adrenal androgen concentrations across sexes.

Among girls, children with exaggerated adrenarche were older at presentation than those without exaggerated adrenarche [8.09 vs. 7.61 years, *p* < 0.001], whereas age at presentation did not differ according to exaggerated adrenarche status among boys. Bone age advancement (BA–CA difference), height SDS, and BMI SDS were comparable between children with and without exaggerated adrenarche in both sexes (all *p* > 0.05).

Children born with LBW had higher serum DHEAS concentrations and more frequently demonstrated exaggerated adrenarche, whereas androstenedione concentrations were comparable between birth weight groups (Table 2).

Serum DHEAS concentrations showed a weak but significant positive correlation with age at presentation (Spearman ρ = 0.294, *p* < 0.001). Sex-stratified analyses demonstrated a positive correlation between DHEAS concentrations and age at presentation in both girls (Spearman ρ = 0.268, *p* < 0.001) and boys (ρ = 0.421, *p* = 0.001).

Birth weight demonstrated a modest but significant inverse correlation with serum DHEAS concentrations. Pearson correlation analysis revealed a negative association between BW and DHEAS levels (r = −0.251, *p* = 0.0005). The association remained significant after logarithmic transformation of DHEAS concentrations (r = −0.213, *p* = 0.003), indicating that lower LBW was associated with higher adrenal androgen secretion in children with PA. BW SDS demonstrated a modest but significant inverse correlation with serum DHEAS concentrations. Pearson correlation analysis revealed that lower birth weight SDS values were associated with higher DHEAS levels (r = −0.210, *p* = 0.009). This association remained significant after logarithmic transformation of DHEAS concentrations (r = −0.201, *p* = 0.013).

Birth weight and BW SDS were inversely associated with serum DHEAS concentrations, and these associations remained significant after logarithmic transformation of DHEAS (Table 3).

In contrast, DHEAS concentrations were not significantly correlated with BMI SDS (ρ = 0.072, *p* = 0.185) or BA–CA difference (ρ = 0.035, *p* = 0.577). No significant correlations were observed between DHEAS concentrations and BMI SDS or BA–CA difference in either sex.

Among girls, DHEAS concentrations, age at presentation, and BA–CA difference did not differ significantly among normal-weight, overweight, and obese individuals (all *p* > 0.05). Height SDS increased progressively across BMI categories (median 0.80, 0.85, and 1.23, respectively; *p* < 0.001). Post-hoc analyses demonstrated higher height SDS values in obese girls compared with both normal-weight (*p* < 0.001) and overweight girls (*p* = 0.003), whereas no difference was observed between normal-weight and overweight girls (*p* = 0.74). Similarly, among boys, DHEAS concentrations and age presentation were comparable across BMI categories (all *p* > 0.05). Height SDS differed significantly according to weight status, increasing from 0.52 in normal-weight boys to 1.27 in overweight boys and 2.14 in obese boys (*p* = 0.012). BA–CA difference tended to be greater in overweight and obese boys than in normal-weight boys, although this difference did not reach statistical significance (*p* = 0.052). Post-hoc analyses revealed that obese boys had higher height SDS values than normal-weight boys (*p* = 0.009), whereas differences between normal-weight and overweight boys (*p* = 0.18) and between overweight and obese boys (*p* = 0.27) were not statistically significant.

### 3.2. Thyroid Characteristics

Mean free T4 concentrations were 15.95 ± 2.31 pmol/L in girls and 16.25 ± 2.03 pmol/L in boys (*p* = 0.437), while mean TSH concentrations were 2.25 ± 1.04 and 2.39 ± 0.97 mIU/L, respectively (*p* = 0.442). Elevated TSH concentrations (>4.5 mIU/L) were observed in 2.8% of the cohort, were present in 3.8% of girls and 5.6% of boys, with no significant sex difference (*p* = 0.953).

Goitre was identified in four girls (1.1%) and was not observed in boys (*p* = 1.000). Among the four girls with goiter, two had positive thyroid autoantibodies consistent with autoimmune thyroiditis, whereas two were antibody-negative and euthyroid, with no specific etiology identified. Thyroid function parameters and the frequencies of thyroid abnormalities did not differ according to LBW status (Table 3). The frequencies of thyroid pathology, goiter, elevated TSH, and thyroid autoantibody positivity were also similar between groups (all *p* > 0.05).

No significant correlations were observed between BMI SDS and thyroid function parameters. BMI SDS was not significantly correlated with free T4 concentrations (Spearman ρ = −0.106, *p* = 0.067) or TSH concentrations (ρ = 0.084, *p* = 0.145).

Thyroid autoantibodies were assessed in a subset of patients and were positive in 21 of 31 tested girls and 3 of 6 tested boys, with no significant difference between sexes (*p* = 0.554). Among all tested individuals, thyroid autoantibodies were positive in 24 of 37 children (64.9%). In the subgroup without a documented indication for testing, thyroid autoantibodies were detected in 21 of 23 children (91.3%).

**Figure 2:**
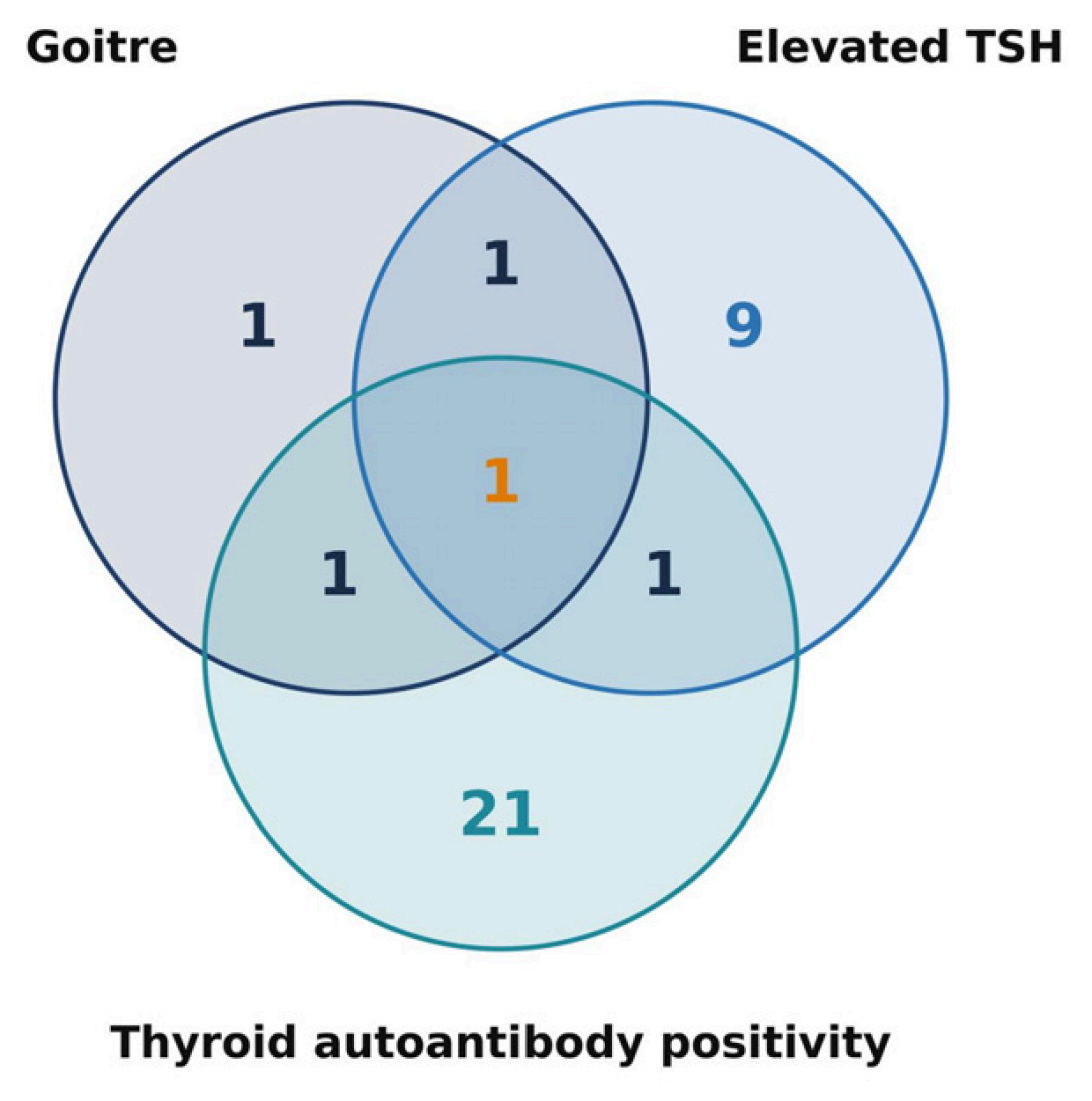
A Venn diagram showing the distribution of thyroid abnormalities. Elevated TSH, positive thyroid autoantibodies, and goitre, and their overlaps in the study cohort. Numbers indicate the count of patients in each region.

Overall, 35 of 435 participants (8.0%) had at least one documented thyroid abnormality, defined as the presence of goitre, elevated TSH concentrations, and/or thyroid autoantibody positivity. The thyroid characteristics of the study population are summarized in Table 4, and the overlap between thyroid abnormalities is illustrated in Figure 2.

### 3.3. Relationship Between Thyroid Status and Adrenal Androgen Profile

Serum DHEAS and androstenedione concentrations were comparable between children with and without thyroid autoantibody positivity [73.9 (43.0–103.0) vs. 78.2 (57.9–139.0) µg/dL, *p* = 0.374, and 0.17 (0.12–0.28) vs. 0.15 (0.12–0.27) ng/mL, *p* = 0.910, respectively].

Likewise, the frequency of thyroid autoantibody positivity did not differ according to exaggerated adrenarche status (69.0% in children without exaggerated adrenarche vs. 42.9% in those with exaggerated adrenarche, *p* = 0.394). Mean free T4 concentrations were similar between children with and without exaggerated adrenarche (15.94 ± 2.24 vs. 16.06 ± 2.24 pmol/L, *p* = 0.722), as were mean TSH concentrations (2.32 ± 0.89 vs. 2.28 ± 1.07 mIU/L, *p* = 0.788).

**Figure 3:**
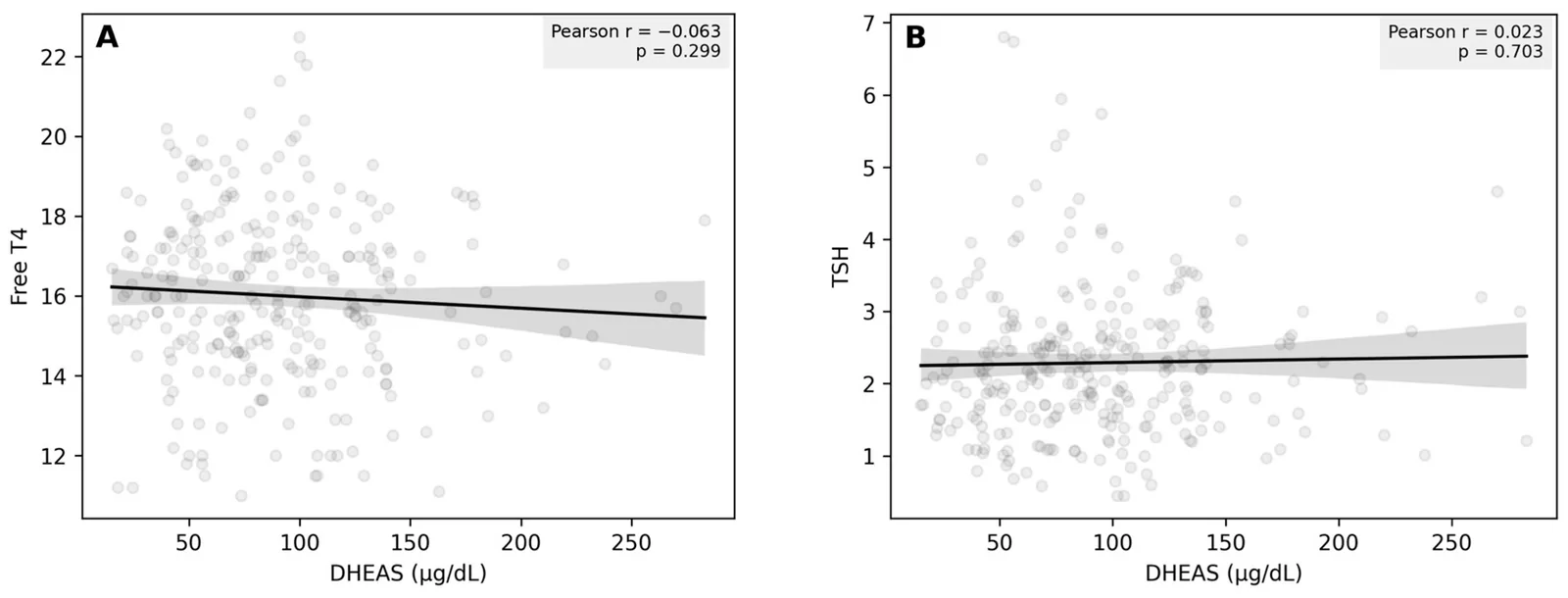
Correlation between thyroid function parameters and serum DHEAS concentrations. (**A**) Scatter plot demonstrating the relationship between serum free thyroxine (fT4) concentration and DHEAS concentration. (**B**) Scatter plot demonstrating the relationship between serum thyroid-stimulating hormone (TSH) concentration and DHEAS concentration. Solid black lines represent Pearson linear regression fits, and shaded areas indicate the 95% confidence intervals. Individual observations are shown as dark gray circles. Pearson correlation coefficients (r) and corresponding *p* values are displayed within each panel.

Correlation analyses further demonstrated no significant associations between adrenal androgen levels and thyroid function parameters (Figure 3). Serum DHEAS concentrations were not correlated with free T4 concentrations (Pearson r = −0.063, *p* = 0.299) or TSH concentrations (r = 0.023, *p* = 0.703). Similar findings were obtained when log-transformed DHEAS concentrations were analyzed.

## 4. Discussion

In this large single-center cohort of children with idiopathic PA, several well-known characteristics of the condition, including female predominance, increased stature relative to parental target height, frequent excess weight, mild advancement of skeletal maturation, and elevated adrenal androgen concentrations, were shown. In addition, we evaluated thyroid disorders and thyroid autoimmunity in this large PA cohort.

Although epidemiological estimates were not a primary objective of the present study, several observations merit attention. First, nearly one-third of children referred with suspected PA ultimately did not fulfill the diagnostic criteria for PA. This finding highlights the substantial clinical burden associated with the evaluation of children presenting with isolated signs suggestive of early androgenization. It underscores the challenges of distinguishing physiological variation from true PA in routine pediatric endocrine practice. Second, the prevalence of NC-CAH among children presenting with PA accounted for 1.4% of cases in our large cohort. Importantly, these data provide real-world evidence from a pre-newborn screening population, demonstrating that evaluation of PA identifies NC-CAH in only a small minority of patients despite its central role in the diagnostic work-up. Previous large cohort studies have reported NC-CAH in approximately 4% of children evaluated for premature pubarche or premature adrenarche [16,17,18]. The lower prevalence observed in our cohort may reflect differences in referral patterns, population characteristics, and diagnostic practices. Nevertheless, our findings confirm that the vast majority of children presenting with isolated PA do not have an underlying adrenal enzymatic defect and instead represent a benign variant of adrenal maturation.

Girls substantially outnumbered boys in our PA cohort, resulting in a female-to-male ratio of approximately 6.6. Similar sex differences have been described previously and may reflect differences in adrenal zona reticularis maturation, adrenal steroidogenesis, or peripheral androgen metabolism [1,19,20]. Interestingly, despite higher circulating DHEAS concentrations, boys did not exhibit more advanced pubic or axillary hair development than girls. This finding highlights the complex relationship between biochemical adrenal androgen secretion and its clinical manifestations. The development of androgen-dependent hair depends not only on circulating adrenal androgen concentrations but also on local intracrine conversion of DHEA and DHEAS to more potent androgens within the pilosebaceous unit, androgen receptor sensitivity, and individual variation in follicular responsiveness. Accordingly, biochemical and clinical markers of adrenarche do not necessarily progress in parallel. Serum DHEAS concentrations increased with age in both sexes, consistent with the physiological maturation of the adrenal zona reticularis during mid-childhood and supporting the biological validity of the study cohort. Notably, boys demonstrated higher DHEAS concentrations and a greater prevalence of exaggerated adrenarche despite representing a minority of patients, suggesting that biochemical adrenal maturation may be more pronounced in boys even when clinical manifestations appear less prominent.

Another important finding was the inverse association between BW and adrenal androgen secretion. Children with lower BW exhibited higher circulating DHEAS concentrations, while those born with LBW were more likely to demonstrate exaggerated adrenarche. These observations align with earlier reports suggesting that restricted fetal growth and an unfavorable intrauterine environment may contribute to earlier adrenal maturation and greater adrenal androgen secretion in childhood [2,21,22]. Potential explanations involve persistent developmental changes in hypothalamic–pituitary–adrenal axis regulation and modifications in zona reticularis development associated with restricted fetal growth. Importantly, this relationship was not confined to the categorical definition of LBW. BW SDS also demonstrated a significant inverse correlation with DHEAS concentrations, suggesting a continuous, dose-dependent association between prenatal growth and subsequent adrenal androgen secretion. Furthermore, age at presentation was comparable irrespective of BW, indicating that the higher DHEAS concentrations observed among children with lower BW cannot be explained by differences in age at presentation. The persistence of this association after logarithmic transformation of DHEAS further supports the robustness of the finding and reinforces the concept that prenatal growth influences adrenal maturation through developmental programming [1,2,23].

Consistent with previous studies, children with PA were taller than expected for their genetic target height and frequently exhibited excess body weight. Previous studies have shown that both premature adrenarche and childhood obesity are associated with accelerated linear growth and advanced skeletal maturation [20,21,22,23,24,25]. In our cohort, height SDS increased progressively across BMI categories in both sexes, whereas DHEAS concentrations remained remarkably similar among normal-weight, overweight, and obese children. These findings suggest that the greater stature observed in obese children with PA is more likely attributable to adiposity-related growth-promoting mechanisms than to the degree of adrenal androgen excess itself [1,26,27,28]. Previous studies provide support for this interpretation, indicating that obesity may promote faster linear growth during childhood through pathways involving insulin, IGF-1, and leptin, with these effects occurring largely independently of pubertal development [1,20].

Serum DHEAS concentrations showed a significant positive association with androstenedione, supporting coordinated adrenal steroidogenesis during adrenarche [1]. The stronger rank-based than linear correlation suggests that the relationship is not entirely proportional across the full biochemical range, likely reflecting interindividual variation in the timing and tempo of adrenal maturation. Together, these findings indicate that DHEAS and androstenedione are complementary rather than interchangeable biomarkers and should be interpreted alongside clinical features, chronological age, pubertal stage, and skeletal maturation.

An important focus of this study was the detailed assessment of thyroid characteristics in a large cohort of children with idiopathic PA. Overall, thyroid abnormalities were uncommon, affecting only 8% of the cohort, and thyroid function remained largely within the normal range. Moreover, neither free T4 nor TSH concentrations correlated with DHEAS levels, and thyroid function did not differ according to exaggerated adrenarche status. These findings indicate that, within our PA cohort, thyroid function parameters were not associated with adrenal androgen secretion. However, because the study did not include healthy control groups, these findings cannot establish whether the observed thyroid characteristics differ from those expected in the general pediatric population or in children with excess weight.

Although experimental and developmental studies suggest biological crosstalk between the thyroid and adrenal axes, our findings do not support a clinically meaningful association within the physiological range of thyroid function [9,10]. Thyroid hormone receptors are expressed within the adrenal cortex, and experimental studies have shown that thyroid hormones influence adrenal development, morphology, and steroidogenic enzyme expression. Clinical observations have also suggested a potential interaction; higher childhood fT4 concentrations have been associated with earlier pubarche, physiological changes in thyroid hormones occur during pubertal maturation, and positive associations between TSH and adrenal steroid concentrations have been reported in pediatric populations [11,12]. Nevertheless, despite these mechanistic and observational findings, our data demonstrate no association between thyroid function parameters and adrenal androgen secretion, suggesting that these interactions may have limited clinical relevance within the physiological range of thyroid function in idiopathic PA.

An additional finding was the high frequency of thyroid autoantibody positivity among children who underwent antibody testing. Because thyroid autoantibodies were measured only in a selected subgroup, this proportion should not be interpreted as the prevalence of thyroid autoimmunity in the overall PA population. Kyritsi et al. reported autoimmune thyroiditis in 26% of euthyroid girls with PA, raising the possibility of an association between PA and thyroid autoimmunity [29]. In healthy pediatric populations, anti-thyroid peroxidase antibody positivity has been reported in approximately 3.4–4.8% of children [30,31,32,33]. In the only available study evaluating Hashimoto thyroiditis in healthy Turkish school children, the reported frequency was 3.6% [34]. Although thyroid autoantibody positivity was frequent among the tested children in our cohort, we found no differences in DHEAS concentrations, androstenedione concentrations, thyroid function tests, or the frequency of exaggerated adrenarche according to thyroid autoantibody status. Importantly, our study did not include a healthy pediatric control group; therefore, these findings cannot establish that thyroid autoimmunity is more frequent in children with PA than in the general pediatric population. Prospective controlled studies with systematic thyroid antibody assessment are required to clarify whether thyroid autoimmunity is truly associated with PA.

The strengths of this study include its large cohort size, the comprehensive clinical characterization of children with idiopathic PA, and the systematic assessment of both adrenal and thyroid parameters within a single-center setting.

Several limitations warrant consideration. First, owing to the retrospective nature of the study, complete thyroid antibody measurements were not available for all participants, which may have resulted in selection bias in the evaluation of thyroid autoimmunity. Second, thyroid antibodies were measured only in a selected subgroup rather than systematically across the entire cohort, precluding accurate estimation of their prevalence. Third, the study did not include healthy control groups. Because an appropriate contemporaneous control group was not available due to the retrospective design, we could not determine whether the observed thyroid findings were specifically associated with PA or reflected background findings in the general pediatric population or in children with excess weight. Fourth, the relatively small number of boys reduced statistical power for some sex-specific analyses. Furthermore, final adult height could not be reliably determined in a sufficiently large proportion of the cohort, given the retrospective and cross-sectional study design. Consequently, the potential long-term influence of idiopathic PA on attained adult height could not be assessed. Longitudinal studies following children with PA until final adult height are needed to clarify its potential impact on long-term growth outcomes.

In conclusion, idiopathic premature adrenarche is characterized by accelerated linear growth, frequent excess weight, and an inverse relationship between birth weight and adrenal androgen secretion. Within this cohort, thyroid function parameters were not associated with adrenal androgen production. Because no control group was included and thyroid autoantibodies were assessed only in a selected subgroup, the present study cannot determine whether thyroid abnormalities or thyroid autoimmunity are more prevalent in children with PA than in the general pediatric population. Prospective population-based studies are needed to clarify this relationship.

## Figures and Tables

**Table 1:** Clinical and biochemical features of children with premature adrenarche.

	Girls (*n* = 378)	Boys (*n* = 57)
**Age at presentation** years	7.8 (7.0–8.4)	8.3 (7.7–9.1)
**Gestational age** weeks	38.0 (37.0–39.0)	38.0 (38.0–39.0)
**Prematurity** *n* (%)	37 (9.8)	6 (10.5)
**Birth weight SDS**	0.5 (−0.1–0.9)	0.4 (0.0–0.6)
**SGA** *n* (%)	18 (4.8)	0 (0.0)
**Height SDS**	0.9 (0.2–1.7)	1.0 (0.1–2.1)
**MPH SDS**	0.1 (−0.6–0.8)	0.0 (−0.8–0.5)
**Height SDS–MPH SDS difference**	0.8 (0.0–1.5)	1.0 (0.5–1.8)
**Bone age**	8.0 (7.0–9.0)	8.5 (7.5–9.0)
**Bone age–chronological age difference**	0.5 (−0.1–1.4)	−0.2 (−0.3–0.3)
**BMI SDS**	0.8 (0.1–1.5)	0.9 (0.1–1.5)
**Obesity** *n* (%)	79 (20.9)	11 (19.3)
**Overweight** *n* (%)	78 (20.6)	13 (22.8)
**Pubic hair** Tanner stage *n* (%)		
**1**	63 (16.7)	9 (15.8)
**2**	237 (62.7)	44 (77.2)
**3**	78 (20.6)	4 (7.0)
**Axillary hair, present** *n* (%)	196 (51.8)	22 (38.6)
**Breast development,** Tanner stage > 2 *n* (%)	63 (16.7)	-
**Testes volume** > 4 mL *n* (%)	-	2 (3.5)
**DHEAS**, µg/dL	85.0 (55.5–115.0)	113.5 (69.7–142.8)
**Androstenedione**, ng/mL	0.26 (0.15–0.38)	0.28 (0.18–0.47)

Data are presented as median (IQR). SDS, standard deviation score; SGA, small for gestational age; MPH, midparental height; BMI, body mass index; DHEAS, dehydroepiandrosterone sulfate.

**Table 2:** Thyroid features of children with premature adrenarche.

Variable	LBW (<2500 g)	Birth Weight ≥ 2500 g	*p* Value
**Age at presentation**, years	7.6	7.8	0.568
**DHEAS**, µg/dL	115.0 (71.6–155.5)	83.1 (54.4–124.0)	0.008
**Exaggerated adrenarche**, %	42.9	22.9	0.028
**Androstenedione**, ng/mL	0.30 (0.16–0.41)	0.23 (0.14–0.38)	0.245
**Free T4**, pmol/L	15.6 (14.5–16.9)	16.4 (14.8–17.6)	0.240
**TSH**, mIU/L	2.48 (1.72–3.02)	2.18 (1.61–2.61)	0.183

Data are presented as median (IQR) or percentage, as appropriate. LBW, low birth weight; DHEAS, dehydroepiandrosterone sulfate; TSH, thyroid-stimulating hormone.

**Table 3:** Correlation analyses of birth weight and DHEAS.

Association	Correlation Coefficient (r)	*p* Value
**Birth weight vs. DHEAS**	−0.251	0.0005
**Birth weight vs. log-transformed DHEAS**	−0.213	0.003
**Birth weight SDS vs. DHEAS**	−0.210	0.009
**Birth weight SDS vs. log-transformed DHEAS**	−0.201	0.013

SDS, standard deviation score; DHEAS, dehydroepiandrosterone sulfate.

**Table 4:** Characteristics according to birth weight status in children with premature adrenarche.

	Girls	Boys
**Goitre**, *n* (%)	4 (1.1)	0 (0.0)
**Free T4**, pmol/L	15.95 ± 2.31	16.25 ± 2.03
**TSH**, mIU/L	2.25 ± 1.04	2.39 ± 0.97
**Elevated TSH** (>4.5 mIU/L), (%)	3.8	5.6
**Thyroid autoantibody positivity** (TPOAb and/or TgAb), *n*/*N* (%)	21/31 (67.7)	3/6 (50)

Data are presented as mean ± SD or *n* (%), unless otherwise indicated. TSH, thyroid-stimulating hormone; TPOAb, thyroid peroxidase antibody; TgAb, thyroglobulin antibody. Thyroid autoantibodies were assessed only in a subset of patients; percentages were calculated using the number of tested individuals as the denominator.

## Data Availability

The datasets used and/or analyzed during the current study are available from the corresponding author on reasonable request.

## Abbreviations

The following abbreviations are used in this manuscript:

ACTH	Adrenocorticotropic hormone
BA	Bone age
BMI	Body mass index
BW	Birth weight
CA	Chronological age
CLIA	Chemiluminescence immunoassay
DHEA	Dehydroepiandrosterone
DHEAS	Dehydroepiandrosterone sulfate
fT4	Free thyroxine
IQR	Interquartile range
LBW	Low birth weight
LC–MS/MS	Liquid chromatography–tandem mass spectrometry
NC-CAH	Non-classical congenital adrenal hyperplasia
PA	Premature adrenarche
PMOS	Polyendocrine metabolic ovarian syndrome
SGA	Small for gestational age
SDS	Standard deviation score
SD	Standard deviation
TgAb	Anti-thyroglobulin antibody
TPOAb	Anti-thyroid peroxidase antibody
TSH	Thyroid-stimulating hormone
WHO	World Health Organization
